# Combined effects of cues influencing patients’ purchasing behavior in online health-care communities: qualitative comparative analysis based on cue utilization theory

**DOI:** 10.1186/s12911-022-02023-0

**Published:** 2022-10-31

**Authors:** Dixuan Ren, Baolong Ma

**Affiliations:** School of Management and Economics, Beijing Institute of Technology, Number 5, Zhongguancun Road, Haidian District, Beijing, 100081 China

**Keywords:** Medical service, Purchasing behavior, Cue utilization theory, Online healthcare communities, Qualitative comparative analysis

## Abstract

**Background:**

The sudden outbreak of COVID-19 in early 2020 pushed the online health-care communities (OHCs) into the public eye in China. However, OHCs is an emerging service model, which still has many problems such as low patient trust and low patient utilization rate. Patients are the users and recipients of web-based medical services, as well as the core of medical services. Thus, based on cue utilization theory, this paper studies combination effect of influencing factors in patients’ purchase of web-based medical services through the qualitative comparative analysis method of fuzzy sets (fsQCA).

**Methods:**

This paper discards statistical methods based on variance theory-based relationships between explanatory and explained variables and uses a construct theory-based fuzzy set qualitative comparative analysis (fsQCA) approach to elucidate such complex relationships of patients' online purchasing behavior. We use a crawler to automatically download information from Haodf.com. This study crawled data in August 2020, involving 1210 physicians.

**Results:**

Service price, reputation and service quality are the key factors for patients’ purchasing behavior. Physician’s online reputation, online medical service price, number of published articles, mutual-help group, and appointment registration affect patients' purchasing behavior by means of weighted variation. Only when a high scope of internal attribute-related cue elements and a low scope of external attribute-related cue elements are combined with each other in a specific form, patients will generate purchase behavior.

**Conclusion:**

This paper clarifies the complex causes that promote to patients' purchasing behavior of web-based medical services, enriches and develops the relevant theories in the field of consumer purchasing behavior and online health-care communities market research, and has implications for governments, platforms, physicians and patients in the event of web-based medical service purchases.

## Introduction

During the new coronavirus epidemic, online health-care communities (OHCs) have been favored and chased by the capital market, and the demand for web-based medical services has seen a blowout-like growth, and with the support of a series of policies, the development of web-based medical services has accelerated. Number of online consultations on several OHCs showed a surge during the epidemic, with the period of Spring Festival 2020, the daily activity of independent APPs in the online consultation sector peaked at 6.712 million people, with a maximum increase of nearly 1.6 million people, or 31.28% [1]. Therefore, 2020 is also known as the "first year of the outbreak" of web-based health care.

OHCs are becoming a valuable platform for patients to communicate and seek support [2, 3], and are providing a new way of developing medical services in China. The patients’ purchasing behavior is the driving force and an important indicator to measure for the development of OHCs. How patients choose online consultation service has always been the focus of many practitioners and scholars.

The influence of physicians’ reputation and word-of-mouth information is mainly considered when exploring patients’ decision to choose online medical services [4–7]. From previous studies, it can be found that from the perspective of medical services, the decision to purchase behavior is a deliberate behavior made by patients through a series of information search and utilization [8, 9], and whether the patient implements the purchase behavior involves an extremely complex scenario, a complex process of interaction among cues, which cannot be explained by a single determinant such as the service quality. The existing literature is biased to examine the influence of single or a few factors on purchasing behavior, and this binary relationship constitutes an analytical idea that is difficult to clearly elucidate the mechanism of patients' behavior and the causes of events, ignoring the impact of multiple elements on patients' purchasing behavior in the actual medical scenario, resulting in a theoretical dialogue among the findings of previous studies that is not convincing enough. Therefore, results of existing studies on the purchase of web-based medical services are fragmented.

At present, the academia a lot of research on patients’ purchasing behavior of web-based medical services, but these studies mostly through regression method or experimental method to get results, the existing research is symmetric causal relationship among variables (the independent variable is the necessary and sufficient conditions of the dependent variable) and “net effect” (excluding other variables, the real effect of a single variable). However, according to the theory of consumer behavior, there are many factors affecting consumer purchasing behavior. Patients’ purchasing behavior in OHCs belongs to consumer purchasing behavior, which is influenced by a series of complex factors such as low-scope cues and high-scope cues, namely combination effect. Based on the above information, this paper takes an aggregated and holistic perspective, and uses the qualitative comparative analysis (QCA) method founded by Larkin to investigate the drivers and antecedent constructs of patients' purchasing behavior [10], using patients who have purchased web-based medical services in China as an empirical study. This paper aims to find out all the paths that can produce these purchasing behaviors, that is, what combination of each influencing factor can lead patients to purchase web-based medical services (combination effect). The complex causes of online purchase activities were clarified by analyzing the influence of the interaction of antecedent elements such as physician title, reputation, and articles on patients' willingness to purchase behavior, and six equivalent outcome constructs of patients' decision to purchase were identified.

The innovation of this paper lies in that it studies the combined effect of patients’ online purchasing behavior based on fsQCA, which expands and deepens the research methods and conclusions in the field of patients’ online purchasing behavior and OHCs. On this basis, this paper focuses on and contrasts and explains the role of these outcome constructs in patients' purchasing behavior, provides an in-depth analysis of the reasons that drive patients' decision to purchase online medical service behavior, and makes targeted recommendations to improve patients' willingness to purchase to help platforms and physicians for using more effective ways to increase sales. It helps medical administration to introduce relevant policies to give full play to the role of web-based medical services. Also, it proposes recommendations to help platforms and physicians, use more effective ways to increase sales, help medical administration to introduce relevant policies, give full play to the substitution and complementary role of online medical services to offline medical care, allocate medical resources rationally and improve the efficiency of consultation. The conclusions of the direct and indirect elemental configurations of medical services drawn in this paper are of great value to correctly guide patients to seek web-based medical treatment and to reasonably match the needs between physicians and patients in the short term.

## Literature review

### Complexity theory

The complexity theory lays a theoretical foundation for exploring the sufficient and necessary conditions and the combined effects of influencing factors of patients’ purchasing behavior in OHCs. According to complexity theory, the relationship among variables in the real word is not necessarily linear or non-linear, and the same factors will have different influences in different situations. In the data analysis of multiple linear regression, although the data can support the positive influence of independent variable X on the result variable Y, there are also case of high X and low Y or low X and high Y in the samples, regression analysis cannot explain these reverse cases. Complexity theory and QCA method point out that the generation of the same result can be caused by multiple paths, that is to say, the combination of different variables can explain the same result variable, so as to help researchers explain these reverse cases. By explaining the complex relationship among variables, complexity theory provides a more powerful tool to explain the mechanism of patients’ purchasing behavior in the context of online medical treatment. Therefore, based on complex theory and with the help of fsQCA method, this paper explores the different paths of patients’ purchasing behavior in OHCs, so as to find out the sufficient and necessary conditions for its occurrence.

Based on the complexity, this paper focuses on the factors that influence the occurrence of patients’ purchasing behavior from both high scope of internal cues and low scope of external cues. According to theory of consumer behavior, there are many factors affecting consumer purchasing behavior. Web-based medical advisory services have the trust property, it is difficult to judge before, during and after consumption. The technical quality of service between physicians and patients has serious information asymmetry. According to the signal theory, patients use cues from physicians’ homepage released to decide whether to buy their services [11].

### Influencing factors of patients’ purchasing behavior

The attributes of product quality are divided into experience quality and search quality [12]. Experience quality means that the buyer can judge the quality of the product only after using the product, and search quality means that the consumer can judge the quality of the product by searching before purchasing the product. As the research progressed, the product attributes were expanded from two categories to three categories, and trust quality was added. The new trust quality refers to the fact that consumers cannot judge the quality information of goods even after they have consumed them. Products are classified as search goods, experience goods, and trust goods based on the quality characteristics of the product attributes. Expert service goods are trust goods, where doctors not only sell services but also participate in the whole process of service realization, including, medical, repair, consulting, education and other fields of expert service goods [13]. The quality uncertainty of trust goods comes from the information asymmetric information between buyers and sellers (asymmetric information), in the trust goods market, consumers are uncertain about what they need, the quantity and the degree of demand, the final purchase of the delivered goods by consumers is determined by the producer, there is a serious information asymmetry between buyers and sellers [14].

A single product or service can have empirical quality attribute, search quality attribute and trust quality attribute at the same time, for example, the taste and texture of food products belong to empirical quality attribute, the production date belongs to search quality attribute, and whether it contains a series of additives such as coloring agents and preservatives belongs to trust quality attribute. The accuracy of information is a trust quality attribute, which is difficult to determine before and after the service, but some quality signals can be used to convey quality cues, such as the price of the service, the online reputation, the grade of the offline hospital where the physician works, and the physician's status [14].

Health care is a high trust-demanding service and patients may face difficulties in not being able to determine the quality of the service and make choices about their available treatment options [15, 16]. Trust, as one of the important guarantees of relational exchange, has a profound impact on the online doctor-patient relationship, so it is necessary to study patient choice behavior in online health communities from the perspective of the cues that may be utilized in the patient's decision-making process. The number of reviews [17], review length [18], price [19], and seller reputation [20] have an impact on sales volume. For the online health consultation market, it was studied that physicians’ response frequency, type of services opened, physician status, degree of information disclosure, and online reputation all had a positive effect on patient purchasing behavior [21]. Website design, security, privacy [9], and website feature application [20] had an impact on sales volume. Table 1 presents related research of patients’ purchasing behavior.

**Table 1:** Related research on patients’ purchasing behavior

Related research	Independent variables	Dependent variables	Results
Yang, Guo, Wu and Ju (2015) [4]	The score of patients and the score of platforms as third party	The number of patients consulting online physicians	The synergistic effect between system-generated information and patient-generated information is positively correlated with the patient’s decision to consult a physician
Zeng and Guo (2018) [21]	Personal attributes and word of mouth	Patients’ behavior to choose their physicians	The frequency of physicians’ reply, the number of services opened, the professional title of physicians, the degree of information disclosure, and the online word of mouth positively affect patients’ behavior of choosing physicians
Liu, Wu and Lu (2017) [22]	Generate patients’ and physicians’ information, generate system information	Patients’ purchasing behavior	The generated information of patients and physicians has a positive influence on patients’ purchase of services, while the generated information of the system has no display effect
Cao, Liu, Zhu, Hu and Chen (2017) [5]	Physicians’ service quality and electronic word-of-mouth(eWOM)	Patients’ selection decisions	Service quality and eWOM both had positive effects on patients’ selection decisions. Disease knowledge increased the importance of service quality on patients’ choice. Disease risk and disease knowledge reduce the influence of eWOM on patient choice
Liu, Guo, Wu and Wu (2016) [7]	Multilevel and cross-level reputation	Physicians’ performance	The number of physicians’ appointments is positively correlated with their individual offline and online reputations, as well as the offline and online reputations of the hospitals where the physicians work
Liu, Li and Ju (2019) [23]	Online service reviews and offline service reviews	Number of telephone consultation	Patients with high-risk diseases were more likely to pay attention to offline patient reviews, while patients with low-risk diseases were more likely to pay attention to online service reviews
Wu and Lu (2017) [24]	Online text consultation, telephone consultation	Offline consulting service reservation	Text consultation was positively correlated with offline service appointment, while telephone consultation was negatively correlated with offline service appointment
Li, Song, Zhao, Guo, Ju and Vogel (2019) [25]	Subjective and objective information in OHCs	Patients are transferred from online to the physician’s offline service	Patients are more likely to switch from online to offline medical services with physicians whose subjective and objective information on their homepages show a higher quality
Shah, Yan, Shah, Shah and Mamirkulova (2019) [26]	Patient-generated signals (PGSs), system-generated signals (SGSs)	Physician choice	PGSs SGSs have a significant impact on patient decision. Disease risk negatively moderates the relationship between PGSs and information search, while disease risk positively moderates the effect of the two signals on patients’ willingness to pay prem

Based on the analysis of previous research [11, 21, 27], we found that although scholars have conducted abundant studies on patients’ purchasing behavior, the conclusions are independent from each other, and the analysis of single factors fails to fully explain the phenomenon of patients’ purchasing behavior. Patients’ purchasing behavior involves many and complex factors, so it is necessary to conduct an integrated analysis on all the influencing factors of patients’ purchasing behavior. To fill this gap, we use the cue utilization theory and fuzzy set qualitative comparative analysis (fsQCA) approach to investigate complex factors of patients’ purchasing behavior. Because the decision-making process of consumers is to process a series of cues, and finally get the purchasing decision, that is, consumers’ purchasing behavior is the result of the combination of factors, rather than the result caused by the influence of a single factor. In this paper, we will select the main factors from both high-scope and low-scope cue elements of medical services to study the causes of patients' purchasing behavior in a holistic and in-depth manner.

### Research model

**Fig. 1:**
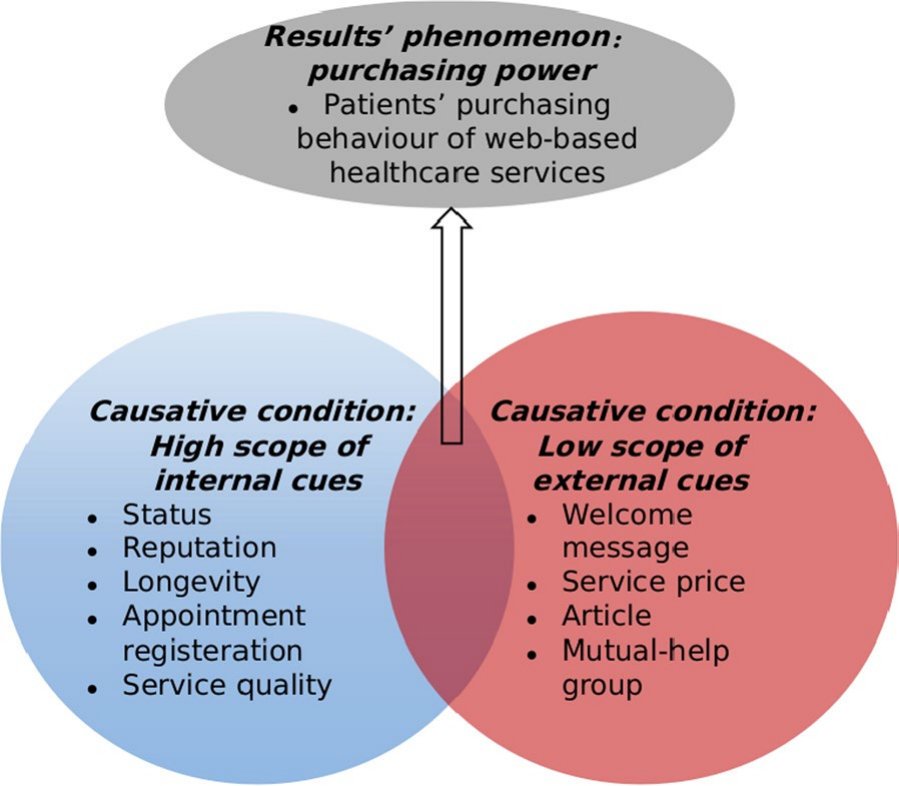
Research model for patients’ purchasing service behavior

The research model of this study is shown in Fig. 1. medical services are a special commodity, and before consultation, patients need to have an understanding of the physician's technical level, medical ethics and their own condition, and the physician's advice may directly affect the patient's degree of recovery or even their lives. The lack of face-to-face consultations has led to a serious information asymmetry between physicians and patients, which requires patients to get to know physicians in depth through the tools provided by OHCs, and constant communication between physicians and patients to build trust. The OHC, as an Internet industry providing medical services, is at the stage of public awareness, where the focus is on cultivating patients' habits and building their trust in the platform and the physician, whose purchasing behavior plays a decisive role in the development of both the platform and the physician. Patients can make judgments about the quality of physicians’ services through the quality signals released by physicians [11]. Based on the trust perspective to study the factors influencing patients' purchasing behavior to physicians in OHCs, the trust transfer effect of high scope internal cues as well as low scope external cues was explored in terms of cue utilization theory to explore patients' influence on doctor selection behavior in OHCs.

## Method

### Analysis method–fsQCA

Qualitative comparative analysis (QCA) is an analysis method for small and medium sample case studies, which emerged in social science research in the 1980s and is mainly applied in sociology, politics and economics [28]. The QCA method tries to integrate the traditional quantitative and qualitative methods, and regards it as a research orientation beyond the two. Its basic logic is based on the principle of Boolean algebra, and explores the common characteristics of multiple cases by discussing the membership relationship among sets. At present, multiple linear regression method is often used in marketing research, which is a research method to study the degree of net impact, which makes scholars focus on testing whether a single variable is statistically significant [29]. On the contrary, the focus of QCA method is not how a single condition variable leads to the occurrence of the result, but how multiple different condition variables combine with each other to jointly affect the final result. In view of these advantages of QCA method, some scholars call for jumping out of the traditional research horizons of qualitative and quantitative research, and applying QCA method to carry out research in the field of economic management [29].

In the initial development of QCA method, the assignment of condition variable and result variable followed the most basic 1/0 division of Boolean algebra, namely QCA method based on clear set (csQCA). However, in the process of coding, some concepts often can neither be completely reduced to 1 nor completely reduced to 0, but are in an intermediate state. Based on this reality, the QCA method has evolved into a comparison method of fuzzy sets (fsQCA) [30], and introduced the concept of membership degree, that is, according to the gap between each condition variable and 1/0, it is assigned to the value of 0–1 interval, and then the fuzzy set algorithm is used to calculate the membership degree value. Since the variables involved in this study could not be simple classified as 1 or 0, fsQCA method was adopted to test the hypothesis in this paper.

**Fig. 2:**
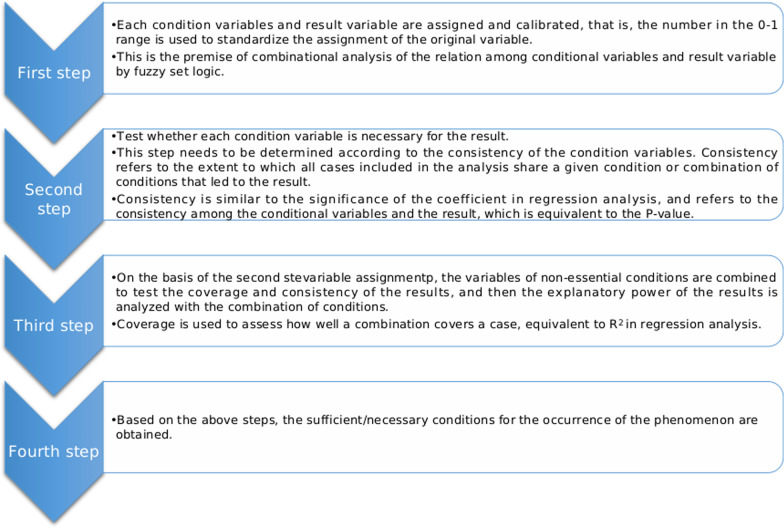
Flowchart of fsQCA

Some variables including longevity, appointment registration, welcome message, article and mutual-help group [13, 31]. Therefore, this paper argues that the effect of cueing alone is not enough to fully reveal the influencing factors of patients’ purchasing behavior in OHCs, and it is necessary to continue to explore the influence of different cueing combinations on patients’ purchasing behavior through combination effect analysis. For example, although the net effect of the article is not significant, its combination with other factors may still influence patient purchasing behavior. Thus, this paper uses fsQCA method to test the combination effect of high-scope internal cues and low-scope external cues, that is, how these factors combine to produce an effect. FsQCA is an emerging research method based on fuzzy set theory, which can conduct a more in-depth analysis of the causes of the results or phenomena. It emphasizes that the result is caused by a combination of factors, and gives a combination of factors that can explain the result, and explores the sufficient/necessary conditions behind the phenomenon. Figure 2 shows the analysis steps of the combined effect.

### Study design

Consumers judge product quality through a series of cues. Due to the information asymmetry between providers and buyers of online medical consultation service [19], patients can judge whether to buy medical services according to the cues reflecting quality signals. The cue utilization theory suggests that the extent to which a cue is used to assess product quality depends on its quality diagnostic properties and the availability of other cues [32]. The diagnostic properties of product cues are classified as low-scope cue and high-scope cue [33]. Low scope refers to cues that are easily perceived as manipulative, which give vague indicators of product quality, e.g., brand name and price; high scope cues are cues that build up over time, which are more reliable and credible, e.g., reputation. Also, Consumers use the extrinsic and intrinsic attributes of integrated products to judge product quality [34]. Extrinsic attributes are not physical components of the good and changes do not materially affect the actual product, but they are often seen as cues that may influence consumer perceived quality, e.g., brand names, warranties, etc. Intrinsic attributes tend to shape evaluation information by influencing extrinsic cues because intrinsic cues are more valuable than extrinsic cues [32]. However, it is only when cues with intrinsic attributes are rare or of little value to utilize that external attributes are more likely to be used for quality assessment of goods [35, 36].

Since welcome message, service prices, articles and mutual-help groups are not service components of online consultation, and are subject to modification by physicians at any time. (1)Physicians will set up a welcome message on their personal pages. The welcome message will introduce the physician’s basic information, professional expertise, and express the hope that patients come to consult. Welcome message can make patients perceive physicians’ professional commitment (such as enthusiasm, responsibility, morality, etc.) [37], so as to influence purchasing behavior. (2)Physicians set price for online services based on individual circumstances. High prices are used to deliver high quality information [31], in other words, patients deem that high prices represent high service quality. (3) Physicians publish medical articles in their areas of expertise on their home page. Patients can read these articles for free. OHCs can effectively guide patients’ behavior, treatment decisions, health expectations and outcomes, and behavioral changes, which are all influenced by the knowledge published by physicians in the community [38]. 4) The physician’s home page in OHCs has been set up a column of mutual-help group that is a collection of virtual discussion groups in which members can share experience, feelings and knowledge about health topics of their interests [39]. Thus, we classify the above variables as low scope of external cues. 

These variables, which include status, reputation, longevity, appointment registration and service quality, are reliable, credible cues that are established over time, and they may influence external cues. (1)Physicians’ status affects patients’ purchasing behavior. Physicians providing online medical services have different ranks, such as chief physician or associate chief physician et al., and the differences in these ranks will result in different status of online physicians. (2)Reputation is the most important cue of product quality [11, 40], thus affecting patients purchasing behavior. Reputation in online marketplaces is largely based on feedback [41]. (3)The reason why customers prefer to patronize century-old store is that the stores with longevity can convey a signal of good service quality to customers. Similarly, the longer a physician’s homepage has operated, the better the quality of his or her service has been recognized by patients [31]. (4)In a physician’s homepage, whether the function of appointment registration is opened will affect patients’ online purchasing behavior. Online consultation mode can initially realize triage and connect between patients and physicians. However, further treatment needs to be carried out offline. Thus, patients will preferentially choose to a physician who open an appointment registration. (5) service quality is the most important internal cue that influences patients’ purchasing behavior. The quality of medical service is related to patients’ health and even life. Thus, the above variables are classified as high scope of internal cues.

High scope of internal cues and the low scope of external cues in Fig. 1 are essential causal conditions that lead patients to purchase online health consultation services. The results may be explained by the formation of multiple different constructs between the cues. The quality of health care services affects patients' perceived value and thus determines purchasing behavior [42]. The specificity of online medical services leads to more complex reasons for the formation of patients' purchasing behavior, the research framework contains more elements, and the impact of changes in single element on purchasing behavior is very limited, so research on whether patients will purchase online medical consultation service products cannot consider only unilateral effects. Only when the high-scope cues and the low-scope cues are combined with each other in a specific form can higher patients’ purchasing behavior be generated.

Thus, we hypothesize:

#### H1

 When the high scope of intrinsic attributes and the low scope of extrinsic attributes appear simultaneously, the purchasing behavior will appear.

There is a causal asymmetry in consumer purchasing behavior, i.e. for a case of purchasing behavior that has occurred, whether a single element plays a facilitating or inhibiting role in it does not depend on the main effect of the variable itself on the outcome, but it depends on its configuration with other variables [44]. Patients are the consumers who purchase online health consultation services. Patients make judgments about the quality of online health consultation services based on cues related to online health services, and online indicators reflecting doctors' competence include physicians' status, reputation, years of online service, online registration and online service quality, all five of which are high-scope internal cues about physicians' service competence and evaluations accumulated through long-term online and offline services for patients. Sales of various products are influenced by the number of online reviews and review scores, and reputation has a greater impact on sales of experienced products [43].

Eleven factors under the dimensions of content, design, security, and privacy of the web page (privacy statement, frequency of updating web page content, providing personal user accounts, aiding decision-making, providing company information, etc.) were studied for their impact on online sales volume [43], and merchant website feature applications had an impact on online sales volume [37]. The content displayed on online physician web pages (word of expressing welcome, service prices, articles, mutual-help group [31], etc.) are cues independent of the product or service, i.e., external attributes, and they can be altered by the physician at any time and, therefore, are also low-scope cues. The psychological behavior of patients in this process is complex and cannot be measured by a simple linear relationship, and there is asymmetry, which leads this paper to propose hypothesis 2 and hypothesis 3.

Thus, we hypothesize:

#### H2

 A single driver element in the set of cause conditions may influence on patient purchase behavior, depending on the way that element and other elements are conformed.

#### H3

There are several different constructs that can generate user purchase behavior, and the elements contained in each construct may be different.

#### Data collection

This study crawled data from haodf.com for analysis. Haodf.com is a typical online health consulting service platform in China that has a long history of development, mature operation, high user coverage and high physician activity [31]. For these reasons, Haodf.com is investigated in this paper. As of June 21, 2022, haodf.com has gathered 10,096 regular hospitals with 890,580 physicians in 31 provinces and cities across China. Thus, Haodf.com is a representative OHCs.

This study crawled data in August 2020, involving 1210 physicians. After excluding records with missing values, there were 13,240 data from 1205 physicians in the end.

## Measures

In this study, observed variables were used to measure the perceived service quality of patients before the onset of purchase behavior, and the patient-perceived quality of health-care services included nine indicators such as physician’s status, reputation, longevity, appointment registration, service quality, welcome message, service price, articles, and mutual-help group. Table 2 shows how each variable was measured.

**Table 2:** Measurement and description of variables

Variables	Description	Measure
Patients’ purchasing behavior	Physicians sell the number of health-care services on haodf.com	Using the original data
Status	Position of the physician in the offline hospital	Dummy variables were used for the metric. Status was categorized into 5 levels, with chief physicians as level 5, associate chief physicians as level 4, attending physicians as level 3, residents as level 2, and other physicians as level 1
Reputations	The reputation of a physician is measured using a combination of 3 metrics: the number of patient votes, thank you letters and gifts. The haodf.com allows patients to vote, write thank you letters and give gifts to physicians. When a patient feels good about physician’s services, he can vote for him, or write a thank you letter, or give gifts to the physician, such as flowers and banners and other virtual items. The more votes, thank you letters and gifts a physician gets, the better the physician's reputation is	Since the three indicators of votes, thank you notes, and gifts have large ranges of values, they were each standardized and then averaged to measure the reputation of the physicians
Longevity	The time that physicians register personal homepage for online medical services	The number of years between the time the physician’s home page was created and the time the data were collected
Appointment Registration	If the physician opens an online registration function on the website, then the patient can reserve an offline appointment with the physician through the online health consultation platform, saving the patient's time in the offline queue for registration	Dummy variables are used for the metric. A "1" is assigned to a physician who is open for appointment registration, and a "0" to a physician who is not
Service quality	Once a patient purchases a medical service on the website, they can consult with the physician on the website and the patient's questions will be answered by the physician on the platform	Number of answers by physicians responding to patient questions online
Welcome message	Physicians open a personal page on the platform, where they can set a prominent place at the top of their personal page to introduce their information as well as their expertise, etc. and express their wish for patients to purchase their medical services	Dummy variables were used for the metric. A welcome message of "1" is set for the physician's personal page and "0" is set for no welcome message
Service price	Purchase price of online text consulting service for physicians	Using the original data
Article	Number of articles published by physicians on the website	Using the original data
Mutual-help group	An online health consultation community is a collection of virtual discussion groups in which members can share their feelings, knowledge and experiences about health topics of interest to them [47]. Physicians divide patients who have purchased their medical services into groups on the platform and place patients with similar illnesses within the same group to facilitate communication between patients	Using the original data

The correlation coefficients between the variables are given in Table 3. From Table 3, it is clear that there is no large correlation between the variables and hence the multicollinearity is not serious.

**Table 3:** Pearson correlation coefficient

Variables	(1)	(2)	(3)	(4)	(5)	(6)	(7)	(8)	(9)	(10)
Patients’ purchasing behavior (1)	1									
Status (2)	. 069	1								
Reputation (3)	. 650**	. 143*	1							
Service quality (4)	. 666**	. 085	. 765**	1						
Price (5)	. 161**	. 109	. 302**	.260**	1					
Welcome (6)	. 015	. 101	. 091	. 069	. 079	1				
Appointment (7)	. 103	. 119*	.225**	. 123*	. 159**	. 067	1			
Longevity (8)	-. 021	. 449**	. 156**	. 125*	. 008	. 128*	. 091	1		
Article (9)	− .004	− . 165**	− . 002	. 078	− .032	. 085	− .008	. 097	1	
Mutual-help group (10)	. 130*	. 152**	. 385**	. 523**	.088	− .038	.010	. 244**	. 098	1

** for *p* < 0.01, * for *p* < 0.05

## Results

Since this study uses qualitative comparative analysis of fuzzy sets, the first step requires initial assignment of variables with the assignment rules shown in Table 4. Since the welcome message and appointment registration use dummy variables of 0 and 1, they do not need to be reassigned. Other variables need to be converted to fuzzy set values.

**Table 4:** Variable assignment rules

Variables	Value before assignment	Value after assignment
Patients’ purchasing behavior	0 ~ 1503	0, 0.25, 0.5, 0.75, 1
Status	1, 2, 3, 4, 5	0, 0.25, 0.5, 0.75, 1
Reputation	− 0.82 ~ 8.26	0, 0.25, 0.5, 0.75, 1
Longevity	1.76 ~ 11.04	0, 0.25, 0.5, 0.75, 1
Appointment registration	0, 1	0, 1
Service quality	464 ~ 84,169	0, 0.25, 0.5, 0.75, 1
Welcome message	0, 1	0, 1
Service price	0 ~ 1200	0, 0.25, 0.5, 0.75, 1
Article	0 ~ 3654	0, 0.25, 0.5, 0.75, 1
Mutual-help group	0 ~ 1651	0, 0.25, 0.5, 0.75, 1

After the variables in the model are assigned values, the data are imported into the QCA software and the fuzzy set module is used to perform operations on the 10,242 data. The software will first perform truth table construction, listing all possible combinations of constructs that may produce the purchase of health-care services, and automatically filter out the antecedent condition constructs that have some sufficiency for the purchase of health-care services based on the input consistency threshold and case frequency threshold (default criteria: consistency threshold of 80% and case frequency threshold of 1), and the filtered truth table is shown in Table 5, where the initial consistency (raw consist) refers to the proportion of this construct that exhibits the characteristics of the outcome variable, i.e., the strength of explanation of the outcome variable, with a value of 1 representing a high level of this element and a value of 0 representing a low level of this element, and data testing revealed that there were no contradictory combinations in the data (i.e., the same combination of antecedent conditions produced different levels of results), allowing for subsequent comparative analysis.

**Table 5:** Truth Table 1

Status	Reputation	Longevity	Appointment	Service quality	Welcome	Price	Article	Mutual-help group	Case number	Initial consistency
1	1	1	1	0	1	0	0	0	1	1
1	1	0	1	0	1	1	0	0	1	1
1	1	1	1	1	1	0	0	0	1	1
1	1	0	0	1	1	1	0	1	1	1
1	1	1	0	1	1	1	1	0	1	1
1	1	0	1	1	1	1	0	0	1	1
1	1	1	0	1	1	1	1	1	3	1
1	1	1	1	1	1	1	1	0	1	0.937
1	1	1	1	1	1	1	0	1	5	0.932
1	0	1	1	1	1	1	0	1	3	0.932
1	0	0	1	0	1	1	0	0	1	0.924
1	0	0	1	1	1	1	0	0	1	0.924
1	1	1	1	1	1	1	1	1	8	0.923
1	0	1	1	1	1	1	0	0	1	0.907
1	1	1	1	1	1	1	0	0	3	0.904
1	0	1	1	0	1	1	0	1	2	0.900
1	1	1	0	1	1	1	0	1	4	0.894
1	0	1	1	1	1	1	1	1	2	0.875
1	0	1	1	0	1	1	0	0	5	0.874
1	0	1	1	0	1	1	1	1	1	0.870
1	0	1	0	1	0	1	0	1	1	0.857
1	0	1	1	0	1	1	1	0	1	0.845
1	0	0	0	0	1	1	1	0	1	0.816
1	0	1	0	1	1	1	1	1	3	0.810
1	0	1	0	1	1	1	1	0	1	0.781
1	0	1	0	1	1	1	0	0	4	0.774
1	0	0	0	1	1	1	0	0	3	0.774
1	0	1	0	0	1	1	1	1	2	0.772
1	1	1	1	1	1	0	0	1	1	0.769
1	0	1	1	0	1	0	0	1	1	0.749
1	0	1	1	0	1	0	0	0	2	0.734
1	0	1	0	1	1	1	0	1	1	0.724
1	0	1	0	0	1	1	1	0	2	0.721
0	0	0	0	1	1	1	0	0	1	0.710
1	0	0	0	0	1	1	0	0	3	0.709
1	0	1	0	0	1	1	0	1	2	0.707
1	0	1	0	0	1	1	0	0	6	0.675
1	0	1	1	0	1	0	1	0	1	0.675
1	0	1	0	1	1	0	1	0	1	0.671
1	0	0	1	0	1	0	0	0	2	0.658
1	1	1	0	1	1	0	1	1	3	0.654
1	0	1	0	1	1	0	1	1	2	0.652
1	0	1	0	0	1	0	1	0	1	0.638
1	0	1	0	0	0	1	0	1	1	0.625
1	0	1	0	1	0	0	0	1	1	0.625
1	0	0	0	0	0	1	0	0	1	0.622
1	0	1	0	0	1	0	1	1	2	0.616
1	0	0	0	1	1	0	0	0	1	0.563
1	0	0	0	0	1	0	0	0	4	0.483
1	0	1	0	0	1	0	0	1	4	0.471
0	0	0	0	0	1	0	0	0	1	0.463
1	1	1	0	1	1	0	0	1	1	0.444
1	0	1	0	0	0	0	0	0	3	0.417
1	0	1	0	0	1	0	0	0	17	0.361
1	0	0	0	0	0	0	0	0	1	0.355
1	1	1	0	1	0	0	0	1	1	0

The complex solutions derived from the preliminary analysis of this paper are shown in Table 6, all the constructs in the results appear higher physician status, i.e. physicians’ status is necessary for patients to generate the purchase of online medical services, indicating that there are patients who are willing to want a high ranking physician for medical consultation, and consumers believe that high status represents a higher quality of service, but this does not mean that as long as the physician has a high ranking title, he or she will be chosen by patients. The combination of physician status with other elements of the constructs needs to be examined simultaneously. The truth table was then reconstructed after moving the physicians’ status out of the module, and another comparative analysis was performed, and the default criteria were still chosen for the comparative analysis of the reconstructed truth table to obtain truth Table 7.

**Table 6:** Preliminary analysis of complex conditional configurations

Cues	Conditions for configuration								
High-scope, internal cues	1	2	3	4	5	6	7	8	9
Status	⬤	✇	⬤	⬤	⬤	⬤	⬤	✇	✇
Reputation		✇		⬤		⬤	⬤	✇	✇
Longevity	✇	⬤	⬤	⬤	⬤	⬤		✇	⬤
Appointment	⬤	⬤	⬤			⬤	✇	✇	✇
Service quality		✇	⬤	⬤	⬤		⬤	✇	⬤
Low-scope, external cues									
Welcome	⬤	⬤	⬤	⬤	⬤	⬤	⬤	⬤	✇
Price	⬤	⬤	⬤	⬤	⬤	✇	⬤	⬤	⬤
Article	✇		✇	⬤	⬤	✇	✇	⬤	✇
Mutual-help group	✇				⬤	✇	⬤	✇	⬤
Consistency	0.951	0.800	0.895	0.952	0.860	1.000	0.897	0.816	0.857
Coverage	0.062	0.186	0.214	0.176	0.274	0.023	0.063	0.036	0.011
Net Coverage	0.024	0.030	0.055	0.007	0.050	0.012	0.034	0.014	0.011
Total Consistency	0.844								
Total Coverage	0.543								

The black circle (⬤) represents a high level of the element, and the fork (✇) represents a low level of the element. Large circles represent core elements (antecedent elements contained in both the simplified and optimized solutions), small circles represent auxiliary elements (antecedent elements present only in the optimized solution), and blanks represent elements whose high level in the configuration does not affect the results

**Table 7:** Truth Table 2

Reputation	Longevity	Appointment	Service quality	Welcome	Price	Article	Mutual-help group	Case number	Initial consistency
0	0	1	1	0	1	0	0	1	1
1	1	1	0	1	0	0	0	1	1
1	0	1	0	1	1	0	0	1	1
1	1	1	1	1	0	0	0	1	1
1	0	0	1	1	1	0	1	1	1
1	1	0	1	1	1	1	0	1	1
1	0	1	1	1	1	0	0	2	1
1	1	0	1	1	1	1	1	3	1
1	1	1	1	1	1	1	0	1	0.937
1	1	1	1	1	1	0	1	5	0.932
0	1	1	1	1	1	0	1	3	0.932
0	0	1	0	1	1	0	0	1	0.924
0	0	1	1	1	1	0	0	1	0.924
1	1	1	1	1	1	1	1	8	0.923
1	1	1	1	1	1	0	0	3	0.904
0	1	1	0	1	1	0	1	2	0.900
0	1	1	1	1	1	0	0	2	0.895
1	1	0	1	1	1	0	1	4	0.894
0	1	1	1	1	1	1	1	2	0.875
0	1	1	0	1	1	0	0	5	0.874
0	1	1	0	1	1	1	1	1	0.870
0	1	0	1	0	1	0	1	1	0.857
0	1	1	0	1	1	1	0	1	0.845
0	0	0	0	1	1	1	0	1	0.816
0	1	0	1	1	1	1	1	3	0.815
0	1	0	1	1	1	1	0	1	0.785
0	0	0	1	1	1	0	0	4	0.782
0	1	0	0	1	1	1	1	2	0.776
0	1	0	1	1	1	0	0	4	0.774
1	1	1	1	1	0	0	1	1	0.769
0	1	1	0	1	0	0	1	1	0.749
0	1	1	0	1	0	0	0	2	0.734
0	1	0	0	1	1	1	0	2	0.726
0	1	0	1	1	1	0	1	1	0.724
0	0	0	0	1	1	0	0	3	0.709
0	1	0	0	1	1	0	1	2	0.707
0	1	0	1	1	0	1	0	1	0.677
0	1	0	0	1	1	0	0	6	0.675
0	1	1	0	1	0	1	0	1	0.675
0	1	0	1	1	0	1	1	2	0.661
0	0	1	0	1	0	0	0	2	0.658
1	1	0	1	1	0	1	1	3	0.654
0	1	0	0	1	0	1	0	1	0.644
0	1	0	0	1	0	1	1	2	0.626
0	1	0	0	0	1	0	1	1	0.625
0	1	0	1	0	0	0	1	1	0.625
0	0	0	0	0	1	0	0	1	0.622
0	0	0	1	1	0	0	0	2	0.558
0	1	0	0	1	0	0	1	4	0.471
0	0	0	0	1	0	0	0	8	0.455
1	1	0	1	1	0	0	1	1	0.444
0	1	0	0	0	0	0	0	3	0.417
0	0	0	1	0	0	0	0	1	0.379
0	1	0	0	1	0	0	0	18	0.359
0	0	0	0	0	0	0	0	1	0.355
1	1	0	1	0	0	0	1	1	0

Afterwards, based on the theoretical hypotheses proposed by the researcher, the fuzzy set module continues with the simple and difficult counterfactual analyses, simplifying the complex configurations screened in the previous step to produce simplified and optimized solutions that form the outcome variables. In contrast to the optimized solution, the simplified solution is based on two classes of counterfactual analysis in which the antecedent conditions are more stable and are not affected by the researcher's setting of the antecedent conditions for the simple class of counterfactuals. Meanwhile, this study involves more variables, and considering the research rigor and analytical ease [44], the simplified solution will be chosen to explain the patients' purchasing behavior in this paper. At this point, the fsQCA analysis process is completed, and this paper will interpret the results of the analysis and test the hypotheses to analyze the causal constructs of patients' purchasing behavior.

## Results

The data results are shown in Table 8, which yielded a total of six conditional constructs. The total coverage of which is 68.52%, in other words, 68.52% of patients purchasing online medical services can be reasonably explained by the outcome constructs of this study, while the total consistency is 74.89%, i.e., all the constructs possess a good level of adequacy. In this paper, we will test the hypotheses line by line based on the results of the simplified solution in Table 8 and analyze the factors that influence patients' purchase of online medical services.

**Table 8:** Conditional constructs of patients’ purchasing behavior (Unit: %)

Cause-effect	Conditional configuration	Coverage	Net coverage	Total coverage	Total consistency	
Patients’ purchasing behavior	Configuration1	Reputation* ~ Mutual-help group	17.35	2.18	68.52	74.89
	Configuration2	~ Longevity*Article	7.44	1.97		
	Configuration3	Reputation*Price	33.99	3.22		
	Configuration4	Price*Appointment	43.83	14.49		
	Configuration5	Service quality*Price* ~ Welcome	4.11	1.97		
	Configuration6	Service quality*Price*Article*Mutual-help group	28.26	4.87		

" ~ " indicates that this element does not occur, "*" indicates that it occurs simultaneously; coverage and consistency indicate the level of necessity and sufficiency, respectively; net coverage indicates the coverage exclusive to that conditional configuration

### Hypothesis 1 validation

In the latter four constructs of Table 8, online service quality and service price are the key elements that constitute sufficient conditions for patients' purchase behavior of online health care services. That is, high-scope cues or low-scope cues of related elements are unilaterally insufficient to generate purchase behavior, and Hypothesis 1 is supported by the fact that high-scope cues need to be combined with low-scope cues with each other to generate higher purchase behavior. Such a result corresponds to the idea that QCA deals with complex problems, where changes in individual factors have a very limited impact on the results, and where the generation of patient purchase behavior for online medical services is the result of a combination of multiple factors.

### Hypothesis 2 Validation

Observing Table 8, it is found that the service price element is present in all of the last four constructs, and the coverage of constructs 3 and 4 exceeds 33%, which shows that in these four constructs, service price can be seen as a key element in generating purchase behavior, but the presence of this element alone is not sufficient to increase patients' purchase of online medical services; in the first two conditional constructs, the presence or absence of service price does not affect the generation of purchase behavior. Reputation, in addition to being a sufficient condition for purchase behavior in conjunction with service price in configuration 3, also replaces service price as a key element in generating purchase behavior when reputation is high.

Also, the low level of mutual-help group in construct 1 but high level in construct 6 suggests that mutual-help group can increase patient purchasing behavior, but it is also possible that low mutual-help group may promote purchasing behavior along with specific factors. While the articles in Table 3 are not significantly associated with purchasing behavior, the articles in Table 8 appear to positively influence patient behavior in purchasing online health services in construct 6. That is, individual elements may have no effect on purchasing behavior depending on how that element is combined with other elements, and hypothesis 2 is supported.

### Hypothesis 3 validation

**Fig. 3:**
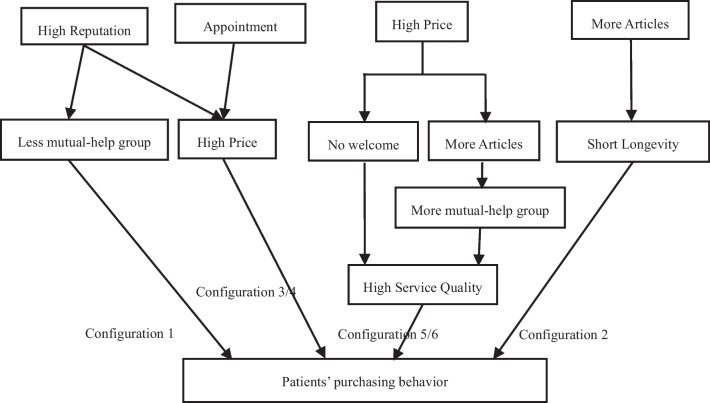
Conceptual pathway

By analyzing the resultant constructs in Table 8, the pathways that form the behavior of patients purchasing online medical services can be derived, as shown in Fig. 3, and this paper will compare and analyze the formation mechanism of each construct pathway and provide an explanation of why patients purchase online medical services in reality. Figure 3 Conceptual pathway for patients to purchase online medical services.

Physicians with higher reputation, configurations 1 and 3, are the paths for that configuration. Physicians with higher reputation will have more online sales when they set up fewer patient groups or have high prices for their services, which corresponds to previous scholarly research. For online marketplaces, reputation is mainly achieved by evaluation feedback systems. Various types of user feedback systems, such as ratings, comments, likes, and gifts of flowers, reflect a feedback from patients about the quality of the service after using the health consultation service. The more satisfied patients are with the quality of the service, the more they tend to give positive feedback such as higher ratings, more positive comments, more likes and more floral gifts. It has been shown that sellers with a better reputation in the review feedback system have a higher price [19]. The more and more positive online patient reviews a physician has, the higher the price premium for that physician's services. This is because physicians with a high reputation have better perceived service quality and, thus, can command a higher price premium. It is noteworthy that fewer mutual-help group or when the service price is high constitutes a high scope of cue elements that increase patients' purchasing behavior, while the level of service quality does not significantly affect patients' purchasing behavior. This suggests that whether users generate purchase behavior in this scenario is not determined by the quality of the physician's service; what users really care about is the good or bad timeliness of the physician's online reputation and the level of charges. The price of the service is the most dominant external quality cue [45]. Price is a direct quality signal given by the physician; price, as the monetary amount proposed by the physician for his or her service, can reflect the physician's assessment of the quality of the service he or she provides, the cost of the service, and for the patient, price is a cost signal as well as a quality signal. High quality promotes sales. This effectively answers the research question of this paper that in some specific scenarios, patients are more concerned about the physician's reputation and the price of the service than the quality of the online medical service; even if the physician works hard online and responds to patients' questions in a timely and detailed manner, patients are unlikely to buy thephysician's product if the physician has a bad reputation.

Physicians opened the online service for not a long time, Construct 2 is the path for this construct. Long time spent in online medical services together with more published articles constitute sufficient conditions for user purchase behavior. The coverage rate for construct 2 was 7.44% and the net coverage rate was 1.97%, indicating that although the number of cases for construct 2 was small, the intrinsic reasons for the formation of user purchase behavior differed significantly from the other constructs. Following the research in cue utilization theory, the number of years of online service is an important factor affecting service uncertainty [19], which in turn affects sales volume. Consumers prefer to patronize century-old stores because the fact that a store can be open for a long time without closing down is itself an indication that it has low service quality uncertainty, but stores that have been open for a long time have also built up over time. All the more reason for early-stage shops to do a better job of promoting themselves. For the online marketplace, the extent of a physician's online efforts is communicated to consumers through the trail of articles. The total number of articles published by the physician affects the user's judgment of the physician's level of expertise and quality of service [19]. Thus, the purchase behavior of configuration 2 is based on the short duration of the physician's online medical services.

Physicians provide appointment registration service, i.e., patients can make appointments online for physicians' offline "face-to-face" outpatient services, and configuration 4 is this configuration path. With the development of Internet medicine, more and more people are aware of this channel to see a physician, and the epidemic has made Internet medicine even hotter. As a new thing, whether online consultation is reliable and how good the physician is, these are also issues that the patients are more concerned about. Most quality physicians have a high volume of offline visits, and it is difficult to get an appointment with that physician at an offline brick-and-mortar hospital. So even if the price of a quality physician's service online is high, patients are willing to buy it because patients can quickly establish a connection with the physician and then reserve an offline visit with the physician online, which can save patients' waiting time in line. Therefore, the higher price of the service is based on the appointment registration.

The types of physicians with higher service prices, configurations 5 and 6, are the paths for that configuration. For the online market, consumers were more concerned about the price of the service than whether the physician's personal homepage had a welcome message. This is consistent with previous research where price is considered to be the monetary consideration for obtaining the use value of a product, or an indication of product quality [46]. The higher the price, the higher the consumer's perceived expected service quality of the good or service [47, 48], and the service provider communicates the level of service through price cues [48]. Online medical services, as trust goods, have serious information asymmetry in the sales process, and price can convey information about service quality to consumers to a certain extent [49], and the higher the price, the higher the quality of the service is likely to be. Although prices are low-scope cues and can be easily manipulated, when consumers do not have sufficient information about product quality, they may take high prices as a signal of quality and choose high-priced goods [50]. Because patients believe that high prices represent high quality, consumers demand a high level of online effort from physicians who not only respond to patients' questions in a timely and comprehensive manner online, but also use more features of the medical platform to provide health information and help to patients, which is the reason why physicians in configuration 6 have to publish more health articles as well as set up patient clubs to build a platform of emotional support for patients.

In summary, hypothesis 3 is supported by the fact that there are six constructs that increase patients' purchase of online medical services, with each purchase behavior path containing some of the same elemental constructs, with construct 3 and construct 4 belonging to the same behavior path and construct 5 and construct 6 belonging to the same path.

### Robustness tests

This study strictly follows the steps in the QCA guidebook for arithmetic analysis, and at the same time, to ensure the stability of the research results, this paper conducts robustness tests in the following two aspects: (1) after moving the necessary conditions in the configurations out of the research model, the conditional configurations of the results remain basically unchanged; (2) after raising the consistency threshold to 0.85 for arithmetic, configuration 2 disappears and the remaining five configurations are the same as the original results. Therefore, the main conformational results obtained by fsQCA analysis in this paper are credibly robust.

## Discussion

### Principal results

This paper dissects the complex causes of patients' online purchasing behavior from a more systematic and comprehensive perspective, and uses fuzzy sets to qualitatively and comparatively analyze the complex dynamic processes of factors such as physician status, reputation, longevity, appointment registration, online service quality, welcome message, service price, articles, and mutual-help group on consumer purchasing behavior in the medical service domain. Based on real data from the Haodf.com, the fuzzy set module was used to identify six equivalent constructs that influence patients' purchasing behavior, and these constructs were generalized and explained. The specific findings are as follows.

- (1) Patient-perceivable high-scope service quality cues will combine with low-scope cues to form sufficient conditions for generating purchase behavior (H1). This suggests that different types of online service cues will generate different purchase behaviors, so in the actual online medical service management activities, it is not enough to unilaterally improve the internal cues of online medical service quality or to focus on external cues only; medical platforms should establish a system that meets the needs of different physicians' success according to their actual situation. For physicians, they should use the functions provided by websites so as to provide patients with more cues expressing their medical competence, i.e., online health-care providers need to examine this issue in an integrated manner by combining elements such as high-scope internal cues with low-scope external cues.
- (2) High-scope intrinsic cues and low-scope extrinsic cues influence patient purchase behavior online through a weighted approach (H2). Consumer purchasing behavior is a complex process and it is difficult to directly explain online patients’ purchasing behavior by a single element; the same level of physician or low scope cue-related factors may increase or decrease the occurrence of purchasing behavior under different cue conditions, depending on the way of conformation with other elements. Thus, studies of user purchase behavior need to examine constructs and such weighting elements in an integrated manner, and it is not meaningful to focus only on the extent to which individual elements explain the results.
- (3) Patients' purchasing behavior for online medical services is formed by six main constructs(H3): constructs 1 and 3, "physicians with a high reputation", which together with less mutual-help group or a high price of services constitute a sufficient condition for purchasing behavior. Construct 2 "physicians with a short time for online medical services", which together with the number of articles published online is a sufficient condition for purchasing behavior. Physicians who offer appointment registration and have a higher price for their services have better online sales, and construct 4 is this type of construct. Physicians with higher service prices and online service quality have higher online sales when they do not have a website welcome message. Construct 5 is a construct of this type. Construct 6 "higher service price", when the physician sets a higher service price, the online service quality and supporting services (more articles and mutual-help group) must also be good to have higher online sales.

### Theoretical and management significances

#### Theoretical implications

This paper enriches and deepens the existing theories and research methods in the field of patients’ purchasing online medical services in the following aspects:

First, fsQCA method is widely used in the research of sociology, politics and economics. This paper applies it in the field of medical marketing to explore the effect of various cues on patients’ purchasing behavior in the context of OHCs by taking its advantages in studying asymmetric causality. This not only enriched the research methods in the field of marketing. Second, compared with the previous linear analysis of online medical service sales, this study highlights the effect of both high-scope and low-scope cues on purchase behavior, and summarizes the pathways to purchase behavior based on the interpretation of six types of constructs. This paper clarifies the complex causes of enhancing patients' online purchasing behavior, provides a new perspective for domestic online medical service research, and enriches and develops theoretical studies related to consumer purchasing behavior and the online health consultation market.

Third, the net effect study in existing literature is not enough to fully reveal the role of each cue in patients’ purchasing behavior, and further study of its combined effect is needed to clarify its role in a more comprehensive and complete way. In order to achieve this goal, this paper adopts fsQCA method to study the combination effect of factors affecting patients’ purchasing behavior, and obtains six combinations of conditions that can affect patients’ purchasing behavior, which also proposes a new research perspective for researchers.

#### Management implications

In the process of medical activities, online health consultation platforms will strive to provide consumers with satisfactory services, but due to the special nature of medical services, the platforms cannot prosper without the contribution of physicians, especially high-quality physician resources [31]. Since medical services are trust goods, in order to eliminate the information asymmetry between physicians and patients, platforms provide physicians with many online functions that convey professional competence and skills, eliminate patients' perceived uncertainty, and thus increase the sales of online physicians' services. The findings of this paper based on patients' behavior in purchasing online medical services provide some theoretical support for the orderly and healthy development of online health consultation services by medical administration, online health platforms, physicians and patients, and the conclusions have certain management implications, as follows.

First, both the administration and the online health consultation platform should note that the formation of online medical service purchasing behavior comes from the synergistic influence of the high scope and low scope cue elements of physicians. OHCs should not only continuously improve the ease of use, but also focus on whether the platform features can effectively deliver positive leads and adopt different incentive systems for physicians with different online service profiles. Moreover, in the operation process, high or low scope of individual elements may increase or decrease patients’ purchasing behavior, and the platform needs to pay special attention to the key elements and weighting elements that shape purchasing behavior and take different measures to target them according to the actual medical scenario of each case.

Second, online physicians need to be fully aware that the way to increase sales of online medical services is to fully utilize the business functions offered by the online platform, as high sales are influenced by the synergy of high-scope and low-scope cues. This study is for physicians to reasonably distribute their limited energy and time in different online tools that can increase online sales. Physicians of the same rank, who are new to Internet health care and have not yet built up their own online reputation, should invest more energy and time in publishing articles that will allow patients to learn more about the physician's expertise and skill level, which in turn will increase sales. If the physician has developed a good online reputation, he or she can set a higher price for the service and can get good sales without using many other tools. If the physician's online reputation has not yet been developed, but high service prices and other high scope leads can work together to increase sales.

Third, the level of physicians in the online health consulting market varies, and patients have to use high-scope and low-scope cues to eliminate information asymmetry so that they can restore their health. Patients should use the evaluation mechanism of the platform to find a physician with a good online reputation and who has opened an online reservation function for online consultation. Patients can first understand their condition and match the physician who matches their condition through the articles posted by the physician, then purchase the physician's online medical services, consult the relevant solutions and quickly establish a connection with the physician. When it is necessary to shift from online to offline consultation, offline consultation can be conducted through the appointment registration function, which can reduce the time spent in the offline queue for registration and effectively balance the allocation of medical resources in China.

### Limitations

This paper has generated some advantageous results; however, there are still certain limitations that must be improved future studies. First, data were taken from one online health-care community, the haodf.com, which is the most acceptable web-based medical consultation platforms [51, 52]; so the generalizability needs to be further tested. Future studies will consider collecting information from several different types of OHCs and empirically testing the model’s findings. Second, the reasons for patients' online purchasing behavior in actual health-care activities are numerous and the influencing factors are complex. This paper only examined the elements of cues presented by physicians in the OHC and did not analyze other elements in detail, such as the type of disease [53–55] and individual characteristics of patients, which can be further explored in future studies.

## Conclusion

Most of the population has basically been used to go directly to physical hospitals for consultation and medical treatment due to the influence of traditional thinking, and has not accepted the emerging Internet health care. However, with the outbreak of the new crown pneumonia epidemic in 2020, more and more people became aware of this channel of accessing medical care, and the epidemic made Internet health care even hotter. Many patients began to realize the role of Internet health care during the epidemic, but still held doubts about whether online visits were reliable and how good the physicians were, which were also of greater concern to patients. But after the patient has purchased online medical service experience, this worry will be eliminated, because the patient can contact the original physician who saw him in the offline hospital. The network platform built by Haodf.com allows the best physicians in the country to go out online and provide services to patients by the physicians themselves. The information asymmetry between physicians and patients can be alleviated by applying the theory of cue utilization. The cues to eliminate information asymmetry can be divided into high-scope internal cues and low-scope internal cues. The government, platforms, physicians and patients should note that the formation of online medical service purchasing behavior originates from the synergistic influence of low-scope and high-scope cue elements.

## Data Availability

Our data can be collected on Haodf.com website (www.haodf.com). It is an opening data.
